# Mixed states vs. pure mania in the french sample of the EMBLEM study: results at baseline and 24 months – *European mania in bipolar longitudinal evaluation of medication*

**DOI:** 10.1186/1471-244X-9-33

**Published:** 2009-06-07

**Authors:** Jean-Michel Azorin, Elodie Aubrun, Jordan Bertsch, Catherine Reed, Stephanie Gerard, Michael Lukasiewicz

**Affiliations:** 1 SHU psychiatrie adulte, CHU Ste Margueritte, Marseille, France; 2 Neurosciences Medical department, Eli Lilly and company, Suresnes, France; 3 Fundacio Sant Joan de Déu, Serveis de Salut Mental, Hospital Sant Joan de Déu, Barcelona, Spain; 4 European Health Outcomes, Eli Lilly and company, Windlesham, UK

## Abstract

**Background:**

To describe the clinical course and treatment patterns over 24 months of patients experiencing an acute manic/mixed episode within the standard course of care.

**Methods:**

EMBLEM was a 2-year European prospective, observational study on outcomes of patients experiencing a manic/mixed episode. Adults with bipolar disorder were enrolled within the standard course of care as in/outpatients if they initiated or changed oral medication for treatment of acute mania. After completing 12 weeks of acute phase, patients were assessed every 3–6 months during the maintenance phase. We present the 24 month results, with subgroup analysis for mixed states (MS) and pure mania (PM). These subgroup analyses are driven by the high proportion of antidepressants prescribed in this cohort.

**Results:**

In France, 771 patients were eligible for the maintenance phase. 69% of patients completed the follow up over 24 months. The mean age was 45.5 years (sd = 13.6) with 57% of women. 504 (66%) patients were experiencing a PM and 262 (34%) a MS at baseline. The main significant differences in MS vs. PM at baseline were: a higher rate of women, and in the previous 12 months, a higher frequency of episodes (manic/mixed and depressive), more suicide attempts, more rapid cycling, fewer social activities and more work impairment. Over the 24 months of follow-up the MS group had a significantly lower recovery than PM (36% vs. 46%, p = 0.006). Overall, 42% of all patients were started on monotherapy and 58% on combination therapy; of those 35% and 30% respectively remained on their initial medication throughout the 24 months. At baseline, 36% were treated with an antidepressant, this proportion remains high throughout the follow-up period, with a significantly higher rate for MS vs. PM at 24 months (55% vs. 27%, p < 0.001).

**Conclusion:**

In this large sample, MS occur frequently (34%), they are more severe at baseline and have a worse functional prognosis than PM. Although antidepressants are not recommended in MS and PM, they were frequently prescribed at baseline and are maintained during the 24 months of follow-up.

## Background

Bipolar disorder is a chronic pathology, the course of which is marked by relapses and remissions. Long-term treatment in this pathology is necessary in order to minimize the risks of relapse and recurrence and also to allow optimal quality of life as well as social and personal functioning [1,2]. Therapeutic options for the treatment of acute manic episodes have widened with the availability of atypical antipsychotics. Some international guidelines [1,2] have been updated with regard to the drug treatment of patients experiencing mania, but there has been no assessment as yet of their application in medical practice.

Mania comes in different forms (pure mania, mixed episode, psychosis and/or hallucination) and follows different patterns of progression (first episode, recurrence, rapid cycling, substance abuse). With such a wide variety of presentations, diagnosis of mixed states (MS) is particularly difficult [3,4]. MS are thought to be under-diagnosed and their prevalence not known to any degree of precision (between 6.7% and 37% depending on the sources) [4,5], due mainly to a lack of a consensual definition [6] and also to misleading symptomatology. The depressed state the patients may present with could be the reason for prescribing anti-depressants, although these are not indicated. The course of MS could consequently deteriorate, including a heightened risk of suicide and substance abuse [7].

A previous analysis of the EMBLEM study highlighted the high proportion of antidepressants initially prescribed, suggesting that an associated depressive symptomatology is frequent. These observations support the case for a separate analysis of mixed states (MS) and pure mania (PM).

The *European Mania in Bipolar Longitudinal Evaluation of Medication *(EMBLEM) study was a prospective, observational study aimed at evaluating long and short term outcomes of patients presenting a manic or a mixed episode and at assessing the clinical and functional outcomes [8-12]. The results presented in this article come from the French cohort over a 24-month follow-up period.

## Methods

### Study design

The *European Mania in Bipolar Longitudinal Evaluation of Medication *(EMBLEM) study is a prospective, observational study conducted since 2002 in 14 European countries (Belgium, Denmark, Finland, France, Germany, Greece, Ireland, Italy, the Netherlands, Norway, Portugal, Spain, Switzerland and the UK), 4 of which (Denmark, Germany, Spain and Switzerland) did not take part in the maintenance phase. This study was approved by regulatory authorities and Ethics Committees in each participating country where required. In France, this study was approved by national medicinal council, CCTIRS (pre-data privacy committee) and CNIL (data privacy committee).

The acute phase of the study lasted for 12 weeks and included 5 post-baseline follow-up visits at 1, 2, 3, 6 and 12 weeks following the onset of antimanic treatment. During the maintenance phase, the patients were assessed at 6, 12, 18 and 24 months. Patients gave their informed consent for taking part in the study and confidentiality was kept in compliance with the law on information and liberty.

### Population

To be included, patients had to be aged 18 years or over, presenting as in- or out-patients for the treatment of acute or mixed mania, and initiating or changing oral medication for mania (except for a change of dosage). Investigators were asked, but not obliged, to include in the study an equal number of patients initiated respectively under olanzapine and any other antimanic treatments (neuroleptics, antiepileptics and/or lithium). Treatment decisions were made prior to the decision to participate in the study and all treatment decisions were left at the discretion of the treating psychiatrist. To be eligible for analysis patients needed to have completed the acute phase of the study and be able to participate in the maintenance phase and to have no missing CGI-BP mania or depression ratings at baseline.

### Measurements and Variables

Baseline data included patient socio-demographic characteristics, psychiatric history, treatments prescribed (previously prescribed, prescribed for the episode, concomitant medications). The mania/mixed and depressive symptomology and severity were assessed by various scales at baseline and during the subsequent visits: the *Young Mania Rating Scale *YMRS [13], the Hamilton Depression Rating Scale (modified version of 5 items, tailored to the depressive symptoms of the manic episode) [14] and the Clinical Global Impression – Bipolar Disorder (CGI-BP) scales – sub-scores of overall bipolar symptoms, manic symptoms, depressive symptoms and CGI-BP for hallucinations and delusions [15]. Diagnosis of mixed state was not reported systematically and a dimensional definition was subsequently used. Patients with a score of over 3 on the CGI-BP-mania and CGI-BP-depression scales at baseline were defined as mixed.

"Simple" remissionwas defined for patients with a CGI-BP overall under 3 for 2 consecutive visits with no relapse between the visits. Recovery was defined for patient experiencing a remission and not dissatisfied with life and social functioning is adequate (i.e. no work impairment, independent accommodation and at least 4 social activities in the previous 4 weeks or with a partner (live-in or not live-in).

Relapse was defined for patients not achieving simple remission and patients meeting one of the 3 following relapse criteria: increase in CGI-BP overall since the last visit, with a score of 4 or more, admission to hospital for an acute episode of bipolar disorder and psychiatrist's opinion. Recurrence was defined for patients in simple remission and who met one of the three previous relapse criteria.

### Statistical methods

Most of the results are given in the form of descriptive statistics. Continuous variables are expressed through means and the standard deviation and a Student test was used for comparison. Categorical variables are expressed as percentages and a χ^2 ^test was used for comparison. Survival probabilities were estimated using the Kaplan Meier method and the log rank test was used for comparison. All the tests were carried out in a bilateral situation and the critical value of alpha was set for p = 0.05. Given that this was an exploratory study we did not correct for multiple comparisons.

## Results

### Population -Socio-demographic data

Of the 795 French patients recruited during the acute phase (equivalent to a third of the European cohort n = 2357), between December 1, 2002 and June 30, 2004, 771 patients were eligible for the maintenance phase. The total population was finally 766 patients (because of 5 missing data concerning the diagnosis) (57% female). At baseline, 504 (66%) PM and 262 (34%) MS were reported. 69% (n = 528) of the patients completed the 24 months of the follow-up period. The proportion of patients who withdrew from the trial was significantly higher among those presenting a mixed episode at baseline (37% vs. 28%, p = 0.006).

The mean age of the population was 45.5 years. Half the patients (n = 380) had received high-school or university education, two thirds (64%) had a partner and a majority (77%) had an independent residence. The only significant difference between MS and PM at baseline was the higher proportion of females in the MS group (69% vs. 51%, p < 0.001) (Table 1).

**Table 1:** Baseline socio-demographic and clinical data of the French cohort (n = 766)

	Pure Mania	Mixed episode	p-value **
		
	n = 504	n = 262	
**Socio-demographic data:**			
Age (Mean ± SD)	45.7 ± 13.9	45.1 ± 13.1	N.S.
Gender:			
Male (n,%)	231 (46%)	73 (28%)	<0.001
Female (n,%)	257 (51%)	182 (69%)	
Educational level (n,%):			
None	8 (2%)	4 (2%)	N.S.
Primary	85 (17%)	37 (14%)	
Junior high school/apprenticeship	156 (31%)	88 (34%)	
Secondary	130 (26%)	69 (26%)	
University	123 (24%)	58 (22%)	
Marital status(n,%):			
No partner	175 (35%)	96 (37%)	N.S.
Living with partner	229 (45%)	121 (46%)	
Not living with partner	99 (20%)	43 (16%)	
Type of residence(n,%):			
Dependent	121 (24%)	56 (21%)	N.S.
Independent	383 (76%)	206 (79%)	

**History:**			
Age at onset of symptom(s): (Mean ± SD)			
Bipolar	30.9 ± 11.1	28.9 ± 11.3	0.036
Manic	32.5 ± 11.8	30.9 ± 12.8	N.S.
Depressive	32.3 ± 11.0	29.4 ± 11.3	0.004
Age at first admission to hospital (Mean ± SD)	32.0 ± 11.7	31.6 ± 12.3	N.S
No. of manic episodes in the past 12 months (including current episode):			
1	305 (61%)	130 (50%)	0.009
> 1	173 (34%)	108 (41%)	
Unknown	24 (5%)	22 (8%)	
No. of depressive episodes in the past 12 months:			
None	246 (49%)	62 (24%)	<0.001
≤ 2	214 (42%)	148 (56%)	
> 2	14 (3%)	30 (11%)	
Unknown	29 (6%)	20 (8%)	
Past suicide attempts (n,%)	32 (6%)	49 (19%)	<0.001

**Current episode:**			
Rapid cycle * (n,%)	55 (11%)	68 (26%)	<0.001

* Rapid cycle: at least 4 depressive or manic episodes in the past 12 months

** p-value with significant threshold of a type 1 error α = 0.05

N.S. = not significant

### Psychiatric history

The mean age of onset of symptoms of a bipolar disorder was 30.2 years, and onset was earlier in the MS group compared to the PM (28.9 years vs. 30.9 years, p = 0.004).

In the 12 months prior to baseline, 37% of all patients had at least one manic/mixed episode. The prevalence and frequency of previous depressive and manic/mixed episodes was higher in the MS population (Table 1). 81 (11%) of the patients had attempted suicide. The MS population showed a higher incidence of suicide attempts (19% vs. 6%, p < 0.001) and rapid cycling (26% vs. 11%, p < 0.001) in the past 12 months.

### Current episode

At baseline, the mean overall score on the YMRS was 26.7, (27.4 for the PM and 25.3 for the MS, p = 0.001) (Table 2). The mean score was 4.7 (± 0.9) for the CGI-BP-mania and 2.6 (± 1.7) for the CGI hallucinations and delusions.

**Table 2:** Severity of the illness at baseline and at 24 months.

	Pure Mania		Mixed episode	
	
	Baseline n = 504	24 months n = 364 (72%)	Baseline n = 262	24 months n = 164 (63%)
Consultation (n,%):				
Out-patient	352 (70%)	354 (97%)	169 (65%)	156 (95%)
In-patient	152 (30%)	9 (2%)	92 (35%)	7 (4%)
Suicide attempt:				
0	461 (91%)*	317 (87%)*	207 (79%)*	121 (74%)*
1	22 (4%)*	32 (9%)*	26 (10%)*	29 (18%)*
>1	10 (2%)*	15 (4%)*	23 (9%)*	14 (9%)*
Substance abuse and dependence (n,%):				
Alcohol	128 (25%)	9 (2%)	70 (27%)	7 (4%)
Cannabis	72 (14%)	8 (2%)	28 (11%)	3 (2%)
Other substances	29 (6%)	2 (< 1%)	25 (10%)	0 (0%)

**Assessment scales:**				
YMRS (mean score ± SD)	27.4 ± 8.8*	. (a)	25.3 ± 9.0*	. (a)
CGI (mean score ± SD):				
CGI-BP Overall	4.5 ± 1.2	2.4 ± 1.6	4.7 ± 0.8	2.5 ± 1.4
CGI-BP Mania	4.8 ± 0.9*	1.9 ± 1.5	4.6 ± 0.8*	2.0 ± 1.3
CGI-BP Depression	1.4 ± 0.5*	1.5 ± 0.9*	3.9 ± 0.9*	2.1 ± 1.2*
CGI-BP Hallucinations	2.7 ± 1.7	1.4 ± 0.9*	2.5 ± 1.6	1.6 ± 1.1*
Frequency of social activities (n,%):				
Never	112 (22%)*	44 (12%)	77 (29%)*	19 (12%)
1 – 4 times	219 (43%)*	150 (41%)	116 (44%)*	91 (55%)
≥ 5 times	171 (34%)*	169 (46%)	69 (26%)*	53 (32%)
Impairment of work activities (n,%):				
None	70 (14%)*	175 (48%)*	18 (7%)*	51 (31%)*
Some impairment	406 (81%)*	166 (46%)*	233 (89%)*	106 (65%)*
Satisfaction with life (n,%):				
Satisfied	160 (32%)*	276 (76%)*	47 (18%)*	96 (59%)*
Neither satisfied nor dissatisfied	138 (27%)*	68 (19%)*	47 (18%)*	37 (23%)*
Dissatisfied	204 (40%)*	20 (5%)*	166 (63%)*	30 (18%)*

(a)The YMRS scale was not assessed at the T10 visit, at 24 months.

* = There is a significant difference between the Pure Mania and Mixed Episode patient groups, threshold α = 0.05.

One patient out of four (25%) took no part in social activities over the four weeks before baseline (29% of the MS and 22% of the PM, p = 0.009). 83% of patients reported work impairment, which was higher for the MS (89% vs. 81%, p = 0.003). 63% of the MS vs. 40% of the PM expressed dissatisfaction with life (p < 0.001).

### Assessment at 24 months (Tables 2 &3)

**Table 3:** Course of the illness over 24 months.

	Pure Mania	Mixed episode
	Over 24 months n = 364	Over 24 months n = 164

**Course:**		
→ Relapse^(1) ^(%, IC95%)	53% [49%, 58%]	57% [50%, 63%]
→ Recurrence ^(2) ^(%, IC95%)	12% [9%, 16%]	19% [13%, 26%]
→ Remission ^(3) ^(%, IC95%)	61% [56%, 66%]	62% [55%, 69%]
→ Recovery ^(4) ^(%, IC95%)	46%* [41%, 51%]	36%* [30%, 42%]

Percentages are based on the Survival distribution (Kaplan-Meier estimations).

(1) Relapse: - Maintenance phase (24 months) only: Patients not achieving remission and if the patient meets one of the 3 following relapse criteria:

- Increase in CGI-BP Total since the last visit, with a score of 4 or more

- Admission to hospital for an acute episode of bipolar disorder

- Psychiatrist's opinion, the patient has experienced a relapse since the last interview

(2) Recurrence: - Maintenance phase (24 months) only: If the patient is in remission and then met one of the three previous relapse criteria.

(3) Remission: Maintenance phase (24 months) only: patient with a CGI-BP Overall under 3 for 2 consecutive visits with no relapse between the visits. If the patient is lost to follow-up, the remission is considered as a missing data.

(4) Recovery: - Maintenance phase (24 months): Patient is in functional remission (remission and not dissatisfied with life) and social functioning is adequate (i.e. no work impairment, independent accommodation and at least 4 social activities in the previous 4 weeks OR with a partner (live-in or not live-in)).

* = There is a significant difference between the Pure Mania and mixed episode patient groups, with a risk α = 0.05.

The mean scores on all the clinical impression scales decreased during the 24-month follow-up period, indicating improving symptoms. Patient outcome over the 24 months was estimated by analyzing survival distributions (Kaplan-Meier estimations). 57% of MS patients experienced a relapse over the 24 months and 53% of the PM (p = 0.328, NS). 19% of the MS experienced a recurrence at 24 months (vs. 12%, NS p = 0.267).

The "simple" remission rate was comparable between the MS patients (62%) and the PM (61%). The recovery rate, however, differed significantly between the PM and MS patients over the 24 months (46% for the PM vs. 36% for the MS, p = 0.006).

Most of the patients (88%) had at least one social activity during the four weeks prior to the last follow-up visit, against 75% at baseline. The proportion of patients reporting impairment in their work activities reduced to 52% (46% in the PM and 65% in the MS p < 0.001). After 24 months, over two thirds (70%) of the patients considered they were satisfied with life (76% of the PM and 59% of the MS, p < 0.001) and only 9% of the patients were still dissatisfied (5% of the PM and 18% of the MS patients), against 48% at baseline.

### Medication

#### Previous medication

At baseline, before the onset of the new treatment, 283 patients (37%) were not receiving any antimanic treatment (Table 4), 290 (38%) were on monotherapy and 193 (25%) on antimanic combinations. Of the antimanic agents, the most commonly prescribed (31%) were anticonvulsants, then typical antipsychotics (29%), atypical antipsychotics (AAP) (14%) and lithium (13%). Compliance was low, with 16% of the patients reporting they were compliant half the time and 6% of patients non-compliant. Concomitant treatments were frequent (antidepressants (36%), benzodiazepines (58%), hypnotics (32%)). The only differences reported between MS and PM were antidepressant prescriptions (53% for the MS vs. 28%, p < 0.001) and benzodiazepines (68% for the MS vs. 53%, p < 0.001).

**Table 4:** Medication at baseline and at 24 months

	Pure Mania			Mixed episode		
	
	Baseline n = 504	Treatment of the episode	24 Months n = 364	Baseline n = 262	Treatment of the episode	24 Months n = 164
**Antimanic treatment:**						
Types of therapy (n,%):						
None	184 (37%)	0	9 (2%)*	99 (38%)	0	15 (9%)*
Monotherapy	191 (38%)	187 (37%)*	180 (50%)*	99 (38%)	132 (50%)*	79 (48%)*
Combination	129 (26%)	317 (63%)*	175 (48%)*	64 (24%)	130 (50%)*	70 (43%)*
No. of drugs related to the treatment (n,%):						
None	184 (37%)	0	9 (2%)*	99 (38%)	0	15 (9%)*
1	191 (38%)	187 (37%)	180 (49%)*	99 (38%)	132 (50%)	79 (48%)*
2	98 (19%)	241 (48%)	145 (40%)*	41 (16%)	103 (39%)	56 (34%)*
3 and +	31 (6%)	76 (15%)	30 (8%)*	23 (9%)	27 (10%)	14 (9%)*
Types of treatment: (n,% Compared to the population)						
Lithium	64 (13%)	70 (14%)	57 (16%)*	36 (14%)	40 (15%)	39 (24%)*
Anticonvulsant	161 (32%)	296 (59%)	187 (51%)	79 (30%)	147 (56%)	72 (44%)
Atypical antipsychotics.	75 (15%)	332 (66%)	212 (58%)	36 (14%)	153 (58%)	85 (52%)
Typical antipsychotics	144 (29%)	163 (32%)	77 (21%)	75 (29%)	67 (26%)	34 (21%)

**Compliance:**						
No prescription	131 (26%)		2 (1%)*	60 (23%)		5 (3%)*
Compliance ≈ 100%	253 (50%)		336 (92%)	147 (56%)		138 (84%)
Compliance ≈ 50%	80 (16%)		20 (5%)	45 (17%)		17 (10%)
No compliance	36 (7%)		5 (1%)	8 (3%)		3 (2%)

**Course over 24 months:**						
Maintains (since baseline) (n,% including LTFup):						
Monotherapy	.	.	84 (45%)*			27 (21%)*
Combination			101 (32%)			35 (27%)
The same treatment			129 (26%)*			42 (16%)*
						
Lithium			33 (47%)			17 (43%)
Anticonvulsant	.	.	121 (41%)*	.	.	38 (26%)*
Atypical antipsychotics.			139 (42%)			48 (31%)
Typical neuroleptics			34 (21%)			12 (18%)
**No. of changes of treatment**: (n,%)						
0	.	.	62 (17%)	.	.	33 (20%)
1			59 (16%)			29 (18%)
2			120 (33%)			63 (38%)
3 and +						

**Concomitant treatments:**						
Antidepressants	139 (28%)*	75 (15%)*	98 (27%)*	138 (53%)*	129 (49%)*	91 (55%)*
Benzodiazepines	267 (53%)*	291 (58%)*	132 (37%)	177 (68%)*	187 (71%)*	74 (45%)
Anticholinergic drugs	44 (9%)	41 (8%)	21 (6%)	21 (8%)	27 (10%)	10 (6%)
Hypnotics (others)	156 (31%)	199 (39%)	78 (21%)	92 (35%)	97 (37%)	43 (26%)
						
Types of antidepressant:						
Monotherapy(AD) (n, %):	134 (96%)	73 (97%)	93 (95%)	135 (98%)	127 (98%)	87 (96%)
IRSS	104 (78%)	56 (77%)	74 (80%)	96 (71%)	91 (72%)	65 (75%)
Tricyclic	25 (19%)	13 (18%)	17 (18%)	21 (16%)	15 (12%)	16 (18%)
Others	5 (4%)	4 (5%)	2 (2%)	18 (13%)	21 (17%)	6 (7%)
Combination of AD (n,%):	5 (4%)	2 (3%)	5 (5%)	3 (2%)	2 (2%)	4 (4%)

Compliance: the patient nearly always complies with/accepts his/her treatment for bipolar disorders (≈ 100%), half the time (≈ 50%)

* = There is a significant difference between the Pure Mania and mixed episode patient groups, threshold α = 0.05.

#### Treatment of the manic/mixed episode

The initiated or changed oral treatment was mostly (58%) a combination of antimanic drugs, especially in the PM patients (63% vs. 50%, p < 0.001). The most commonly prescribed drugs for the episode were AAP (63%), typical antipsychotics (30%) and lithium (14%). Two thirds of the AAP (n = 311) were prescribed in combination, the majority with an anticonvulsant (53%). The AAP + lithium combination was common in the MS (16% vs. 11% for the PM).

Prescriptions of antidepressants and benzodiazepines were common, with 27% of patients taking antidepressants (15% of the PM and 49% of the MS, p < 0.001) and 62% taking benzodiazepines (58% of the PM and 71% of the MS, p < 0.001).

#### Treatment over 24 months

Over the 24-month period, only a third (31%) of the population had no change in treatment (34% in the PM, 24% in the MS). Including the 31% of patients lost to follow-up over 24 months, 35% of the patients who had initiated a monotherapy at baseline maintained it over the period (45% for the PM, 21% for the MS, p < 0.001), and 30% of the patients initiating a combination also maintained it (32% for the PM and 27% for the MS, p = 0.168). Over the 24-month follow-up period, 39% of the patients who had initiated an atypical antipsychotic treatment maintained it, 36% for anticonvulsants, 45% for lithium and 20% for typical antipsychotics. 35% of the patients taking antidepressants at baseline maintained the treatment over the 24 months. The proportion of patients taking atypical antipsychotics was stable (56%) over the study period. The AAP + Lithium combination was common in the MS group (28% vs. 18%), while the AAP + anticonvulsant combination was frequent in the PM (56% vs. 39%). Lithium was prescribed more frequently in the MS group (24% vs. 16%, p = 0.025).

Antidepressant prescriptions were stable and common in both groups (55% for the MS and 27% for the PM, p < 0.001). Prescriptions of anxiolytics decreased slightly over the 24 months, with 39% of patients treated with benzodiazepines at 24 months.

## Discussion

The French cohort of the EMBLEM study followed over 24-months showed considerable use of antimanic combinations and concomitant treatments, in particular antidepressants. Mixed states were common and their illness prognosis was poorer compared to the pure mania patients.

Analysis of the EMBLEM cohort provides information on the follow-up, treatment and course of bipolar disorder over the 24-month period. With six out of ten patients in remission during the follow-up period, EMBLEM provides confirmation that long-term treatment of bipolar disorders brings lasting attenuation of bipolar symptoms. Following the difference observed between the rates of relapse and the rates of recurrence, it could be thought that once stabilization has been achieved, antimanic drugs are relatively efficacious in the long term, with lower rates of recurrence.

### Characteristics of mixed states

The prevalence of MS at baseline in the French cohort of EMBLEM was 34% (n = 262), in line with two other cohorts EPIMAN [5] and EPIMAN-II-Mille [16] (respectively 37% and 30%), which used a different categorical definition, but higher than in the European EMBLEM cohort (24%). It could be considered that, in France, psychiatrists are more attuned to identifying the associated depressive symptoms.

The study confirms the characteristics that were previously associated with MS in studies involving small populations (except for the EPIMAN-II-Mille study): patients are predominantly female [5,16], with a higher risk of suicide [7,16], earlier appearance of bipolar symptoms, a higher occurrence of manic episodes and depressive episodes in the past 12 months [9] and rapid cycling [17]. Comorbidity related to substance abuse and dependence commonly identified in the literature [4,16,18,19] was not found; although there was no standardized instrument for addiction screening in the present study.

At 24 months, mixed state patients had a lower recovery rate than pure mania patients (36% vs. 46%, p = 0.006), with a comparable "simple" remission rate. This observation highlights the lasting nature of the functional issues associated with MS (significantly higher work impairment and dissatisfaction with life) and a residual clinical symptomatology showing significantly higher CGI-BP-depression and hallucination scores and more attempted suicides. The course of MS seems worse than in PM patients, with higher rates of recurrence and 1.5 times more MS experiencing recurrence, although these failed to reach significance.

These observations highlight the importance of systematic screening in mania co-occurring with depressive symptoms [4] and a specific course of care.

The observations also suggest, that for certain patients, the persistent nature of residual symptoms can have an effect on bipolar patients' quality of life and predispose relapse [20].

### Medication

#### Antimanic treatment combinations

Treatments initiated for pure mania and mixed states in this study were characterized by a high proportion of combinations throughout the 24-month period, although less than one patient out of three maintained the same combination. Guidelines for treating an acute manic episode, however, recommend initiating monotherapy [1,2]. The number of mixed state patients with a poorer prognosis and which are harder to stabilize could partly explain the high proportion of treatment combinations.

The study design suggested including an equivalent number of patients taking olanzapine and other oral antimanic drugs. Analysis of the medications prescribed does not, therefore, give a precise picture of current practice, especially as regards the use of the various antimanic drugs, but it can offer a better understanding of prescriptions of combinations with atypical antipsychotic agents. AAP agents are frequently prescribed in combination with anticonvulsants or lithium, with a higher proportion of AAP + anticonvulsants in PM patients and AAP + lithium in MS patients. A number of studies have highlighted a limited efficacy of lithium in MS patients [1,19,21]. Combinations of AAP and Lithium or Valproate are recommended for severe forms in several guidelines [1].

#### Concomitant treatments

One of the important outcomes of this study was the frequent use of antidepressants and this was also seen in the European cohort [22]. Similar prescription patterns of antidepressants in mania have already been reported [16,23].

The use of antidepressants, however, is not indicated [1,2] in the treatment of manic or mixed episodes, due to a risk of inducing mood switches [23] and rapid cycling [21]. It is possible that in this cohort, the high occurrence of MS explains the high rate of prescription of antidepressants as much as the high rate of prescription of antidepressants can explain the transition to an MS.

A number of explications can be put forward for the level of antidepressant prescriptions, with in first position the frequent co-occurrence of depressive symptoms with manic states. Three times more antidepressants are prescribed for an MS episode than for a PM episode. Mixed-state episodes do not by themselves, however, explain the total number of prescriptions. 15% of PM patients were prescribed an antidepressant for the initial treatment of their episode, and more than one out of four over the 24 months. There are other reasons relating to why the physician prescribed or maintained an antidepressant, such as the continuation of a prescription for a previous depression, misgivings concerning a brutal discontinuation of antidepressants, anxiety concerning a depressive switch, the patient's insistence on maintaining his/her treatment and also certain psychopathological hypotheses which consider that mania constitutes a depressive equivalent which needs to be treated, etc.

Benzodiazepines (BZD) are also commonly prescribed, both initially and over the long term, especially for MS patients. BZDs are used when treating an acute episode of agitation [1,2], but they are not recommended for long-term use. The brutal and disconcerting mood swings in the mixed states can contribute to the anxiety frequently experienced in such cases and can explain the high prescription levels [16,18,24]. For patients suffering from associated anxiety disorders, certain guidelines recommend using an atypical antipsychotic over the long term [2].

### Study limitations

This was a prospective observational study, which is subject to the usual biases related to this kind of study, in particular observation biases. The effect of the study design, which requested investigators to include an equivalent number of patients prescribed olanzapine and those taking other antimanic drugs, was discussed above in the analysis of prescription patterns.

The post hoc dimensional definition of mixed states in the study is an important limitation that needs to be factored into the interpretation of the results. Although the inclusion criteria specified that mixed states should be included in the same way as pure mania episodes, MS diagnosis was not reported separately. The definition chosen is likely to include states that are significantly different to pure mixed states, especially as regards states associating depressive and manic symptoms to varying degrees, such as dysphoric mania or mixed depressions [4,6,17]. However, the chosen CGI-BP-mania and depression thresholds (>3) were strict enough for most of these states to be considered as pure mixed states. This post hoc analysis is important in that it confirms the frequency of a clinically-significant associated depressive symptomatology in mania and measures the effect on the course and treatment patterns of bipolar disorder.

## Conclusion

In the EMBLEM study, mixed states are common and their progression patterns are generally more severe. Screening of mixed states remains a major therapeutic issue and it is essential to systematically test for mixed-state items during an acute episode. In addition, the reasons for the high level of prescriptions of antidepressants both initially and long-term still need to be further investigates and should be considered as part of the current debate concerning the long-term use of antidepressants in bipolar disorders and of international guidelines which proscribe their use in manic and mixed episodes.

## Competing interests

Jean-Michel Azorin has undertaken consultancy work for Lilly, Aventis, Janssen, Lundbeck, Astra Zeneca and BMS; he has received honoraria and hospitality from Lilly, Janssen, Lundbeck, BMS, Pfizer and Novartis in relation to conference presentations.

This study was sponsored by Eli Lilly and company. Catherine Reed, Michael Lukasiewicz and Elodie Aubrun are currently working for Eli Lilly.

## Authors' contributions

JMA and IG were expert advisors for France in the EMBLEM study so have participated in the study design and drafted the manuscript. JB has performed the statistical analysis. ML and EA drafted the manuscript. CR has reviewed the manuscript. All authors read and approved the final manuscript.

## Pre-publication history

The pre-publication history for this paper can be accessed here:


